# Supplementary figures and images for: Investigating epigenetic biomarkers of age, sex, and disease in captive South African cheetahs (Acinonyx jubatus jubatus)
Source: PLoS One. 2026 Jan 13;21(1):e0336127. doi: 10.1371/journal.pone.0336127 (PMC12798976; doi:10.1371/journal.pone.0336127)

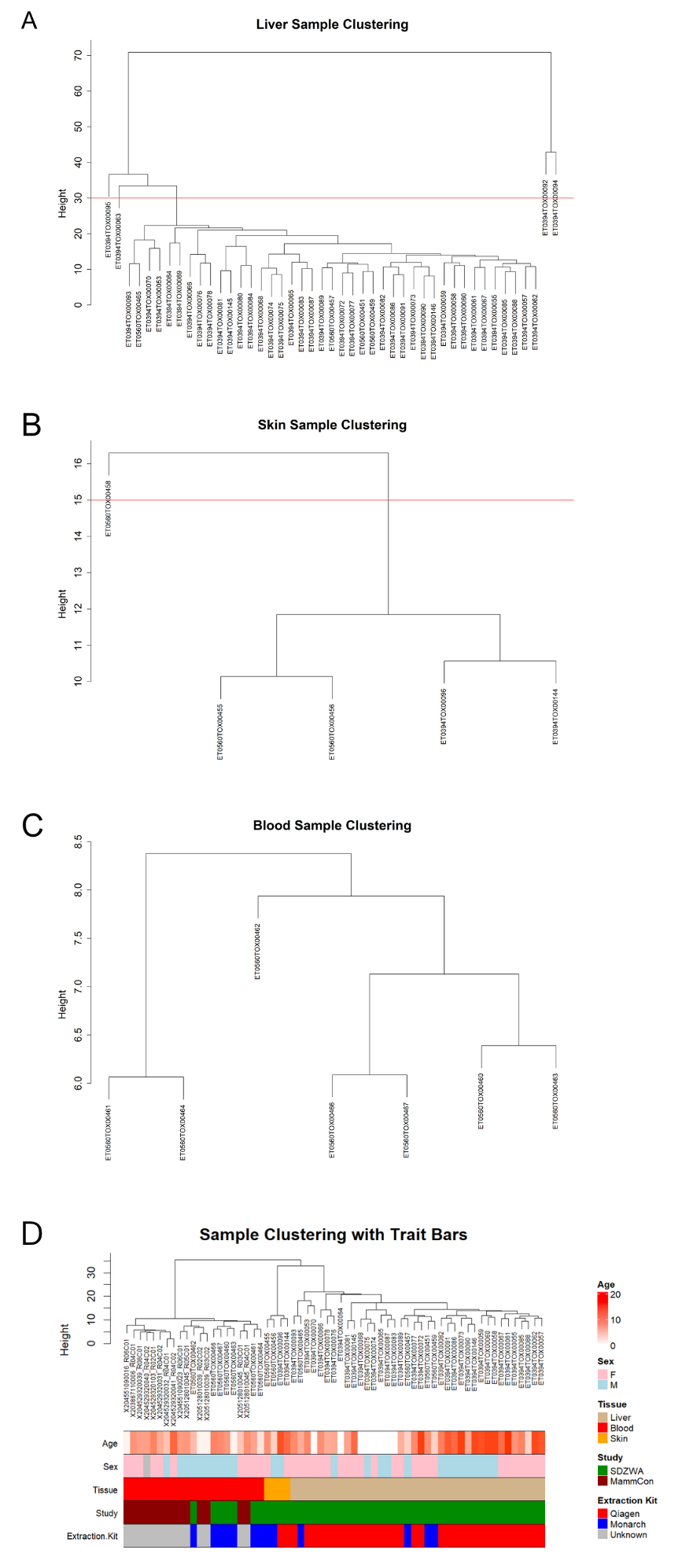

Supplement: S1 Fig — Unsupervised hierarchical clustering was performed to detect outliers in methylation profiles for A) liver, B) skin, and C) blood samples. The red lines in each dendrogram represent the cutoff heights, chosen based on the point of distinct separation in the clustering structure. Samples that clustered separately above this cutoff were identified as outliers and excluded from further analysis. D) Unsupervised hierarchical clustering of all cheetah samples used (outliers removed with the trait bars for annotation. Age spectrum is portrayed in red, with lighter red indicating younger age and darker red indicating older age. Sex is portrayed in pink and blue, with pink indicating female and blue indicating male. Tissue is depicted in red, orange and tan, with red indicating blood, orange indicating skin, and tan indicating liver. Study is portrayed in green and dark brown, with green indicating samples from SDZWA and dark brown indicating samples from MCDB. The DNA extraction kit used is indicated in blue and red, with blue indicating Qiagen extraction kit, red indicating NEB Monarch extraction kit, and grey indicating unknown (MCDB samples). (TIFF) [file pone.0336127.s001.tiff]

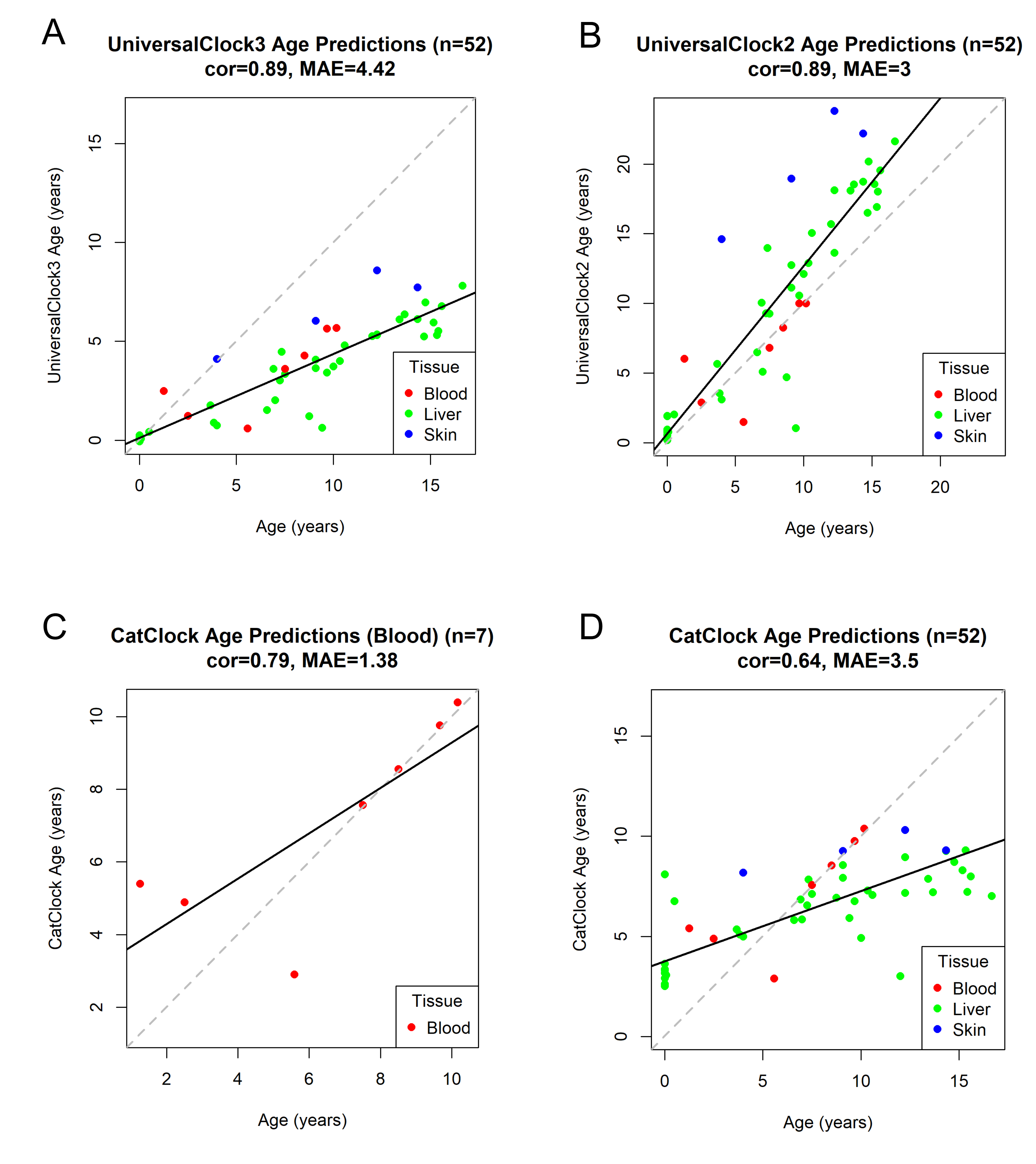

Supplement: S2 Fig — Evaluation of Universal Pan-mammalian clocks on SDZWA cheetah methylation profiles. A) PanClock3 predicted age versus chronological age for cheetah blood, liver and skin samples. B) PanClock2 predicted age versus chronological age for cheetah blood, liver and skin samples. C) UniversalClock2Skin predicted age versus chronological age for cheetah skin samples. D) CatClock predicted age versus chronological age for cheetah blood samples. E) CatClock predicted age versus chronological age for cheetah blood, liver and skin samples. (TIF) [file pone.0336127.s002.tif]

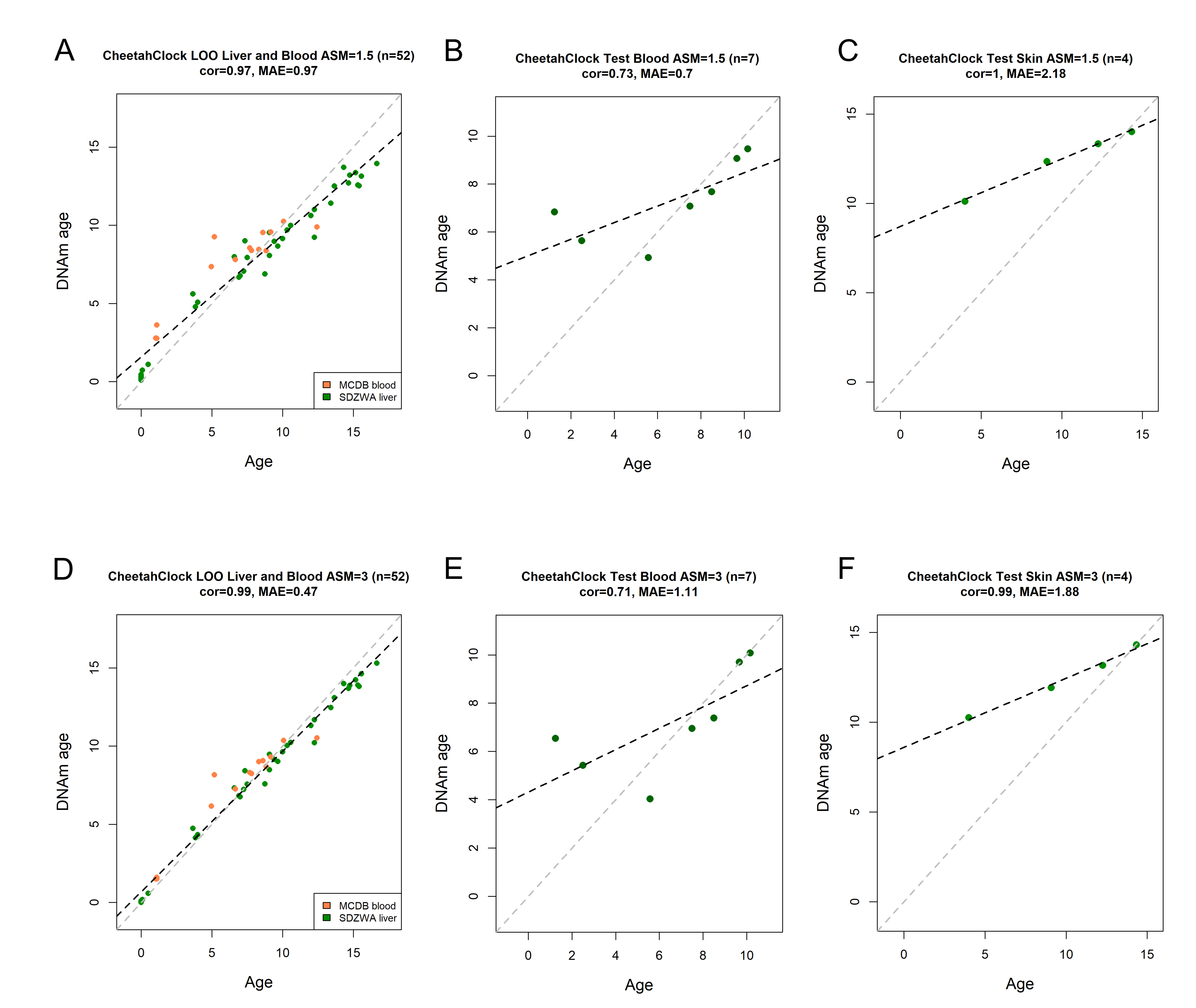

Supplement: S3 Fig — Evaluation of different ages of sexual maturity (ASM) on CheetahClock prediction results. A-C) Evaluation of ASM = 1.5 on A) training liver and blood samples, B) test blood samples, C) test skin samples. D-F) Evaluation of ASM = 3 on A) training liver and blood samples, B) test blood samples, C) test skin samples. (TIF) [file pone.0336127.s003.tif]
