# Supplementary material for: Investigating epigenetic biomarkers of age, sex, and disease in captive South African cheetahs (Acinonyx jubatus jubatus)
Source: PLoS One. 2026 Jan 13;21(1):e0336127. doi: 10.1371/journal.pone.0336127 (PMC12798976; doi:10.1371/journal.pone.0336127)
Supplement: Attachment — Submitted filename: Revision_PlosOne_CheetahEpigClock.docx [file pone.0336127.s017.docx]

**Reviewer´s Comments for manuscript submitted to PLOS ONE**

The manuscript entitled “Investigating epigenetic biomarkers of age and health in South African cheetahs (Acinonyx jubatus jubatus)” presents an important and timely study on the development of an epigenetic clock for cheetahs, a valuable tool for wildlife conservation and management. The authors have generated compelling results showing clear age prediction across different tissues and have extended the analysis to explore biomarkers for sex and Sterile Osteosclerotic Sclerosis (SOS). The study is a significant contribution to the field of conservation genomics. While the core findings are strong, the manuscript would be substantially improved by addressing several key areas. The primary concerns relate to the overall narrative structure, the justification for the experimental design, the discussion of the study's limitations (particularly the use of captive animals and a human-based microarray), and the need for greater methodological detail. The following suggestions are intended to help the authors strengthen the manuscript for publication. I recommend Major Revisions.

Strengthen the Central Narrative and Hypotheses:

The manuscript currently presents analyses of age, sex, and SOS disease, but the biological rationale connecting these variables is not fully developed. The manuscript would benefit from a clearer articulation of the overarching research questions or hypotheses at the end of the Introduction. This would help frame why these specific variables were chosen and what the expected outcomes were, thereby improving the manuscript's logical flow and structure.

Title Revision: The current title does not fully reflect the scope of the investigation. "Health" is a broad term, whereas the study specifically investigates the disease SOS. Furthermore, the use of exclusively captive individuals is a critical detail for interpreting the results, especially those related to biological age. I suggest revising the title to be more specific, for example: “Investigating epigenetic biomarkers of age, sex, and disease in captive South African cheetahs (Acinonyx jubatus jubatus)”.

Applicability and Limitations of Using Captive Animals: The study's reliance on captive individuals is a significant limitation when considering the application of this clock to wild populations. The Discussion should more explicitly address this. Please discuss how factors unique to captivity (e.g., diet, stress levels, veterinary care) might influence epigenetic aging compared to wild counterparts. A more robust discussion is also needed on the challenges of accounting for accelerated aging in wild individuals whose life histories are unknown.

Justification for Genomic Methods: The study utilizes the HorvathMammalianMethylChip40, which was designed for the human genome. While useful for cross-species comparisons, the cheetah genome has been well-sequenced. The authors should justify the use of the human chip and, more importantly, validate their findings. I strongly recommend mapping the identified CpG sites to the cheetah reference genome to confirm their locations and assess the homology of any associated genes between humans and cheetahs. This would significantly strengthen the biological interpretation of the results.

Data Availability: A link to the raw data is missing. For transparency and reproducibility, which is a key policy for PLOS ONE, please provide a link to a public repository where the data are available.

Specific Comments

Abstract:

Please remove non-standard abbreviations from the abstract to adhere to journal guidelines.

L24: I suggest changing the terminology from “over the age of maturation” to a more standard term like “adult” individuals, and specifying the age threshold used (e.g., age > 2 years).

L30-33: The connection between these lines and the preceding sentences is unclear. Please revise to improve the flow of logic.

Introduction:

L44: Please specify whether the epigenetic modifications mentioned correlate positively or negatively with age in this context.

L44-46: For clarity and conciseness, I suggest removing the phrase “and commonly occurs in regions of DNA where these cytosine residues are..”.

L47: Please edit "methyl groups" to the standard form "methyl-groups".

L48: Please clarify what is meant by “structural changes” in this context.

L49: A reference is missing.

L51: Please define what is meant by "Pan-species" upon first use.

L61-62: I suggest rephrasing “tools” to something more precise, such as “these traditional age-estimation methods are difficult to apply accurately”.

L63: Please add "Measurements of" or "The study of" before "epigenetic aging" for logical clarity.

L68: It is crucial information that all samples are from captive individuals with known chronological age, sex, and health status. Please state this clearly and early in the Introduction and Methods, as it is currently difficult for the reader to ascertain.

The Introduction is missing an explicit mention of sex as a variable being investigated. Please incorporate this.

L75-81: This section is the ideal place to formulate the clear hypotheses or research questions mentioned in the Major Revisions.

Methods:

L83: It would be very helpful to state explicitly at the beginning of this section that "All animals were born and raised in captivity and held under human care until death," or similar phrasing.

L89: Please change “die” to “died”.

L91: Please clarify if n=7 refers to the number of samples or individuals.

L93-94: I suggest rephrasing; "depicts correlations" could be changed to "indicates repetitive sampling from the same individuals over time" if that is the intended meaning.

L103: Since two different extraction kits were used, please confirm whether a potential batch effect was investigated and controlled for.

L123: The description of the methods here is too brief; "converted using bisulfite sequencing and put through their previously published pipeline" is not well phrased and insufficient. Please provide a more thorough explanation of the steps involved.

L142: How was the sequencing performed, and how many samples were included in this step?

L148: Please state the significance threshold (e.g., p-value) used for the analysis.

L153: Please consider adding a supplementary table listing the sample IDs, their known health status (especially for SOS), and other relevant metadata for the samples used in the final analyses.

L155-156: The sentence “SOS has been diagnosed in populations as early as 5 years old” is unclear. Does this refer to cheetah populations in general? Please rephrase for clarity. Also, please clarify how the age of these animals was determined.

L156-157: The current phrasing implies causality ("To capture potential epigenetic changes that lead to SOS..."). This is a strong claim. I suggest rephrasing to such as: “To identify potential epigenetic changes associated with SOS…”

L158-165: This section describes various SOS states. For clarity, please focus only on the states that were actually included and analyzed in your study.

L183: Please specify which software or online tool was used to perform the KEGG pathway analysis.

Results:

L189-196: This section appears to describe methods rather than results. Please consider moving it to the Methods section to maintain a clear distinction.

L196-199: Please clarify why stillborn individuals are mentioned if they were not included in the analysis, or remove the mention if it is not relevant.

L202: Please provide the justification for excluding outliers from the analysis.

L204: Please define "pan-tissue" in this context.

L216: Do the "blood" samples refer to whole blood or a specific fraction (e.g., PBMCs)? If whole blood was used, please discuss the potential confounding effect of variable blood cell composition, particularly in the context of disease or infection.

L231: Please use the abbreviation LOOCV consistently after it is first defined.

L246-248: In the text, please specify the sample sizes for each group in this analysis (e.g., N=x males, N=y females; N=x liver, N=y blood).

L249: Please revise the section title to be more descriptive of the results found.

Discussion:

L310: Please ensure the terms "hypomethylation" and "hypermethylation" are used consistently throughout the manuscript and that it is always clear which group is being referenced (e.g., "hypomethylated in SOS-positive individuals compared to controls").

L401: This is a good point. Please expand the discussion of accelerated aging in the context of the cheetah's life history traits.

L409: Suggest changing “under” to “before”.

L411: The lines state, that “Methylation dynamics are often exponential.” Please elaborate on how the results presented in this study support this statement.

L412-417: The observed differences around the age of sexual maturation (ASM) could potentially be confounded by the sex of the animals. Please consider and discuss this possibility.

L425: Were skin samples taken from the same anatomical region on all animals? Different body parts may be exposed to different levels of environmental factors and could exhibit different aging rates. Please clarify or discuss this as a potential limitation.

L507: The sentence ending is incomplete ("hypomethylated in xx"). Please complete it.
