# Supplementary material for: Investigating epigenetic biomarkers of age, sex, and disease in captive South African cheetahs (Acinonyx jubatus jubatus)
Source: PLoS One. 2026 Jan 13;21(1):e0336127. doi: 10.1371/journal.pone.0336127 (PMC12798976; doi:10.1371/journal.pone.0336127)
Supplement: Attachment — Submitted filename: Response to Reviewers.docx [file pone.0336127.s019.docx]

We thank the reviewers and editors for their thoughtful and constructive feedback. We have made revisions to improve the clarity and justifications of our study. Major changes include: (1) refining the title and Introduction to more clearly reflect the focus on captive cheetahs and the investigation of age, sex, and disease-related biomarkers; (2) removing the unused stillborn samples (n = 3) from the total count, and updating in-text tables and S1 Table to reflect this; (3) explicitly stating the study’s hypotheses and strengthening the rationale linking these outcomes; (4) justifying the use of the HorvathMammalMethylChip40 by referencing its cross-species design and supplementing our CpG annotations with cheetah and domestic cat genomic information (S11 Table); and (5) addressing study limitations related to tissue sampling, blood cell composition, and the applicability of findings to wild populations. We also reorganized and expanded the Methods and Results sections, including: (1) editing section titles to be more descriptive; (2) adding a section in Methods to better describe the clustering-based analysis used for outlier exclusion; and (3) updating S1 Fig to include trait bars annotating relevant sample metadata for interpretability. In the manuscript text we incorporated reviewer-recommended references and updated supplementary material titles and descriptions. The renamed supplementary files were re-uploaded to match PLOS ONE style requirements. All edits are described in detail below in red text with line numbers referencing the revised manuscript.

We are currently working to obtain the necessary permissions to upload the raw IDAT files, SESAME-normalized data from The Clock Foundation, and the sample metadata sheet to NCBI GEO. These files will be made publicly available upon approval, and we will notify the editors with the accession number once it has been issued.

Thank you again for your time and consideration.

Sincerely,

Michelle Ysrael, Steven Kubiski, and Caroline Moore

**—------------------------------------------------------------------------------------------------------------------------**

**Reviewer´s Comments for manuscript submitted to PLOS ONE**

Appropriate references should be cited to support the description of the liver disease—veno-occlusive disease (VOD), also known as sinusoidal obstruction syndrome (SOS)—in cheetahs. Notably, SOS is relatively common in captive cheetahs but occurs much less frequently in free-ranging individuals. This distinction should be clearly acknowledged, as it limits the applicability of the epigenetic clock developed in this study to wild cheetah populations. The following references are relevant and should be cited to support this point:

[https://doi.org/10.1177/030098588802500107](https://track.editorialmanager.com/CL0/https:%2F%2Fdoi.org%2F10.1177%2F030098588802500107/1/010f0197780e1f58-72b32737-b9c7-4211-903e-e460c82d1297-000000/ZfNVu-YlaNsDUsgKVc6CfpU35r6X9AYl-FmSBWOmli4=215)

[https://doi.org/10.1638/2020-0084](https://track.editorialmanager.com/CL0/https:%2F%2Fdoi.org%2F10.1638%2F2020-0084/1/010f0197780e1f58-72b32737-b9c7-4211-903e-e460c82d1297-000000/dwwfbe-F_o4eQczC8QHyaVZD8dpIOWN8qt666SkdGHw=215)

- Thank you for these relevant references (reference #23 and #24 in our list) . We cited these studies in the following lines: Line 83, Line 523, and Line 525.
- In our discussion, we currently have stated: “One clinically significant condition in captive cheetahs is sinusoidal obstruction syndrome (SOS), characterized by idiopathic fibrosis targeting vessels that can lead to vascular occlusion, necrosis, and potential liver failure[[23,25,59,60]](https://paperpile.com/c/mdfZo3/aHZLl+IV3h4+3gZKH+aKTM6). While studies have focused on captive populations, SOS has also been observed in wild cheetahs, as well as in other species like snow leopards and humans, though the exact causes in cheetahs remain unclear[[23,24,61,62]](https://paperpile.com/c/mdfZo3/5SSBH+5mVV2+uI9bA+aKTM6).” (Lines 520-525)
- In our conversations with pathologists who have studied this disease, it was pointed out that it is not accurate to say it occurs less frequently in wild populations because the prevalence is not known– it was more accurate to say it is less documented.
- To highlight that this study did focus on captive (and not wild) cheetahs, we added “zoo-housed” to describe cheetahs in the SOS study in the introduction: “For this second goal, we focus on sinusoidal obstructive syndrome (SOS), a recognized disease of **zoo-housed** cheetahs that can be subtle or subclinical in early stages[[22–24]](https://paperpile.com/c/pmRb9u/feVU8+rCEU6+p9p8J).” (Line 83)
- We also added to the age-related discussion: “**Because the model was developed using samples from zoo-housed individuals, its accuracy in wild populations may be influenced by differences in diet, stress and veterinary care which could affect epigenetic aging. Additional testing in wild individuals with known or estimated ages will be important to evaluate and calibrate for these potential differences.**” (Lines 464-468)

The authors should provide a clear justification for the use of a human-based methylation array for cheetahs, particularly given the availability of species-specific genomic resources. To enhance the biological relevance and interpretability of the findings, the authors should evaluate the homology of associated genes between cheetahs and humans and confirm the genomic positions of the identified CpG sites by mapping them to the cheetah reference genome. This additional analysis would provide stronger support for the study’s conclusions.

- The mammalian methylation array (HorvathMammalianMethyl40 Chip) was developed from human and mouse methylation arrays, but was redesigned by Arneson et al [(Arneson et al. 2022)](https://paperpile.com/c/Q4vu58/GBuF) using a 100-way alignment of 99 vertebrate genomes with the human genome to identify highly conserved CpG sites. To highlight this, we added to the introduction: “**Arrays used to build these clocks, including the HorvathMammalMethylChip40 developed by Arneson et al[14], were specifically designed for cross-mammalian application using conserved CpG sites selected through multi-species alignments** ” (Lines 52-55)
- We edited Supplemental Table 11 (annotations of significant CpGs from the SOS DMA) to include both cheetah (Aci_jub_2) and domestic cat (FC.9.0.100) annotations in addition to the original human (Hg38) annotations. The description for Supplemental Table 11 was edited to reflect this: “Results from the differential methylation analysis (DMA) conducted on SOS-positive samples. Includes DMA results for significant CpGs (p.adj < 0.05) found in the analysis: log-fold change (logFC), p-value (P.Value), and adjusted p-value (adj.P.Val), as well as human**, cheetah and domestic cat** gene annotations for each CpG site and CpG location.” (Line 827)
- These annotations were part of the cheetah annotation sheet that accompanied the manifest file from The Clock Foundation. Of note: these cheetah annotations were from the 2018 reference genome Aci_jub_2 RefSeq (GCF_003709585.1) which is currently suppressed for genome annotation processing. We believe providing both the feline and cheetah gene annotations alongside the human annotations will enhance the interpretability of our results and support future homology-based analyses.

Journal requirements:

When submitting your revision, we need you to address these additional requirements.

1. Please ensure that your manuscript meets PLOS ONE's style requirements, including those for file naming. The PLOS ONE style templates can be found at

<https://journals.plos.org/plosone/s/file?id=wjVg/PLOSOne_formatting_sample_main_body.pdf> and

[https://journals.plos.org/plosone/s/file?id=ba62/PLOSOne_formatting_sample_title_authors_affiliations.pdf](https://track.editorialmanager.com/CL0/https:%2F%2Fjournals.plos.org%2Fplosone%2Fs%2Ffile%3Fid=ba62%2FPLOSOne_formatting_sample_title_authors_affiliations.pdf/1/010f0197780e1f58-72b32737-b9c7-4211-903e-e460c82d1297-000000/eDJxDURjBv4Uybkwt0qf3BMUgA-301Yc4u9A3zSpUro=215)

- Supplementary files were re-uploaded to match PLOS ONE style, and renamed in the manuscript: “Supp Figure 1” was renamed to “S1 Fig”, and so on for the other two supplementary figures. “Supp Table 1” was renamed to “S1 Table”, and so on for the other 10 supplemental tables. This was updated in the text:
  - “Supplementary Table 1” changed to “S1 Table” (Lines 106-107)
  - “Supplementary Figure 1” changed to “S1 Fig” (Line 146)
  - “Supplementary Figure 2” changed to “S2 Fig” (Line 217, Line 219, Line 221)
  - “Supplementary Figure 3” changed to “S3 Fig” (Line 235)
  - “Supplementary Table 2” changed to “S2 Table” (Line 236)
  - “Supplementary Table 3” changed to “S3 Table” (Line 241)
  - “Supplementary Table 4” changed to “S4 Table” (Line 242)
  - “Supplemental Table 5-6” changed to “S5 and S6 Tables” (Line 256)
  - “Supplemental Table 7” changed to “S7 Table” (Line 258)
  - “Supplementary Table 8” changed to “S8 Table” (Line 267)
  - “Supplementary Table 9” changed to “S9 Table” (Line 269)
  - “Supplementary Table 10” changed to “S10 Table” (Line 271)
  - “Supplementary Table 11” changed to “S11 Table” (Line 318)
  - Naming of supplemental materials was revised in the same manner at the end of the manuscript (Lines 759 - 821)

2. Please include a complete copy of PLOS’ questionnaire on inclusivity in global research in your revised manuscript. Our policy for research in this area aims to improve transparency in the reporting of research performed outside of researchers’ own country or community. The policy applies to researchers who have travelled to a different country to conduct research, research with Indigenous populations or their lands, and research on cultural artefacts. The questionnaire can also be requested at the journal’s discretion for any other submissions, even if these conditions are not met. Please find more information on the policy and a link to download a blank copy of the questionnaire here: <https://journals.plos.org/plosone/s/best-practices-in-research-reporting>. Please upload a completed version of your questionnaire as Supporting Information when you resubmit your manuscript.

- This form has been filled out and uploaded to Supporting Information.

3. In the online submission form, you indicated that your data will be submitted to a repository upon acceptance. We strongly recommend all authors deposit their data before acceptance, as the process can be lengthy and hold up publication timelines. Please note that, though access restrictions are acceptable now, your entire minimal dataset will need to be made freely accessible if your manuscript is accepted for publication. This policy applies to all data except where public deposition would breach compliance with the protocol approved by your research ethics board. If you are unable to adhere to our open data policy, please kindly revise your statement to explain your reasoning and we will seek the editor's input on an exemption.

- Our team is currently working to obtain the necessary permissions to upload the raw IDAT files, SESAME-normalized data received from The Clock Foundation, and the associated sample metadata sheet to NCBI GEO. We will provide the accession number once it becomes available.

Reviewer #1: This study on methylation changes in cheetahs is novel and valuable, primarily due to its utilization of a unique and novel dataset: biobanked samples from the San Diego Zoo. The availability of precise age and health records for these zoo housed animals is a significant strength. The authors successfully developed a highly accurate epigenetic clock specifically for cheetahs.

Interestingly, the study also presents a novel felid epigenetic clock applicable to both tigers and lions. Further, the authors introduce a new methylation based predictor of sex. A particularly exciting aspect of this research is the exploration of methylation analysis utility in informing disease investigations in zoo housed cheetahs, with a specific focus on sinusoidal obstructive syndrome. Although the latter analysis is only a pilot study, it will be of interest to many readers. The statistical analysis appears robust. The article is well-written but I suggest to add some citations, as detailed below.

I have only minor comments.

1) Optional Suggestion: I wonder if the accuracy of the felid clock could be enhanced by incorporating blood methylation samples from domestic cats. Such data are available through the Mammalian Methylation Consortium. I recommend citing the following article, which presents a clock for domestic cats: K. Raj et al. (2021). GeroScience. Available at: <https://pmc.ncbi.nlm.nih.gov/articles/PMC8599556/>

- We did initially consider using the domestic cat samples in our clock study, but ultimately decided that since most of the domestic cat samples were neutered, they were not representative of intact wild and zoo-house felids such as cheetahs. This was supported by clustering analysis of the cheetah and domestic cat samples: we observed distinct clustering based on sex in the domestic cat samples (Species trait bar = purple), while the cheetah samples (Species trait bar = blue) did not display such distinct sex-based clustering:
-

2) Line 135: Please add a citation to Horvath (2013, Genome Biology) after the sentence "Log-linear age transformation was performed to account for 'accelerated aging' methylation patterns observed before age of sexual maturity (ASM)." This citation is appropriate as Horvath's work introduced the log-linear transformation for human epigenetic clocks.

- A citation to this paper was added (reference #34): “ Log-linear age transformation was performed to account for “accelerated aging” methylation patterns observed before age of sexual maturity (ASM)[**[34]**](https://paperpile.com/c/pmRb9u/aA8S), as done by Lu et al[[35]](https://paperpile.com/c/pmRb9u/CbpRI) in building the pan-mammalian epigenetic clocks, and by Peters et al[[33]](https://paperpile.com/c/pmRb9u/WtvHJ) in building bottlenose dolphin epigenetic clocks.” (Line 157)

3) Data Availability Statement: It would be desirable to deposit the data on the Gene Expression Omnibus (GEO) webpage, including the relevant genomic platform number=GPL28271, to ensure wider accessibility.

- Our team is currently working to obtain the necessary permissions to upload the raw IDAT files, SESAME-normalized data received from The Clock Foundation, and the associated sample metadata sheet to NCBI GEO. We will provide the accession number once it becomes available.

The manuscript entitled “Investigating epigenetic biomarkers of age and health in South African cheetahs (Acinonyx jubatus jubatus)” presents an important and timely study on the development of an epigenetic clock for cheetahs, a valuable tool for wildlife conservation and management. The authors have generated compelling results showing clear age prediction across different tissues and have extended the analysis to explore biomarkers for sex and Sterile Osteosclerotic Sclerosis (SOS). The study is a significant contribution to the field of conservation genomics. While the core findings are strong, the manuscript would be substantially improved by addressing several key areas. The primary concerns relate to the overall narrative structure, the justification for the experimental design, the discussion of the study's limitations (particularly the use of captive animals and a human-based microarray), and the need for greater methodological detail. The following suggestions are intended to help the authors strengthen the manuscript for publication. I recommend Major Revisions.

Strengthen the Central Narrative and Hypotheses:

The manuscript currently presents analyses of age, sex, and SOS disease, but the biological rationale connecting these variables is not fully developed. The manuscript would benefit from a clearer articulation of the overarching research questions or hypotheses at the end of the Introduction. This would help frame why these specific variables were chosen and what the expected outcomes were, thereby improving the manuscript's logical flow and structure.

- A clear hypothesis that addresses the two main study goals was added to the last sentence of the introduction: “**We hypothesized that DNA methylation-based models could accurately predict age and sex in cheetahs, even with a limited sample size, and that incorporating multiple tissue types and related felid species could enhance predictive power and generalizability. Additionally, DNA methylation has been studied for its utility as a biomarker in various human and animal diseases**  [[25,26]](https://paperpile.com/c/pmRb9u/JJobB+yZNWA)**, and we hypothesized that similar epigenetic differences would distinguish SOS-affected and unaffected cheetahs.**” (Lines 84-89)

Title Revision: The current title does not fully reflect the scope of the investigation. "Health" is a broad term, whereas the study specifically investigates the disease SOS. Furthermore, the use of exclusively captive individuals is a critical detail for interpreting the results, especially those related to biological age. I suggest revising the title to be more specific, for example: “Investigating epigenetic biomarkers of age, sex, and disease in captive South African cheetahs (Acinonyx jubatus jubatus)”.

- To better reflect the study scope, edited the title to: “**Investigating epigenetic biomarkers of age, sex, and disease in captive South African cheetahs (Acinonyx jubatus jubatus)”** (Lines 4-5)

Applicability and Limitations of Using Captive Animals: The study's reliance on captive individuals is a significant limitation when considering the application of this clock to wild populations. The Discussion should more explicitly address this. Please discuss how factors unique to captivity (e.g., diet, stress levels, veterinary care) might influence epigenetic aging compared to wild counterparts. A more robust discussion is also needed on the challenges of accounting for accelerated aging in wild individuals whose life histories are unknown.

- Added: “CheetahClock predictions from LOOCV and test samples demonstrate its robustness for cheetah age prediction across tissues, including clinically available samples like blood and skin. **Because the model was developed using samples from zoo-housed individuals, its accuracy in wild populations may be influenced by differences in diet, stress and veterinary care which could affect epigenetic aging. Additional testing in wild individuals with known or estimated ages will be important to evaluate and calibrate for these potential differences.** While further validation with field-curated samples is needed, this is a great advancement for aging these felines in the wild that may potentially replace current expensive and invasive methods of cheetah age approximation such as radiograph analysis of skull morphology and tooth pulp cavity analysis [[17]](https://paperpile.com/c/CGxUM2/sD7sP). ” (Lines 464-468)

Justification for Genomic Methods: The study utilizes the HorvathMammalianMethylChip40, which was designed for the human genome. While useful for cross-species comparisons, the cheetah genome has been well-sequenced. The authors should justify the use of the human chip and, more importantly, validate their findings. I strongly recommend mapping the identified CpG sites to the cheetah reference genome to confirm their locations and assess the homology of any associated genes between humans and cheetahs. This would significantly strengthen the biological interpretation of the results.

- The mammalian methylation array (HorvathMammalianMethyl40 Chip) was developed from human and mouse methylation arrays, but was redesigned by Arneson et al [(Arneson et al. 2022)](https://paperpile.com/c/Q4vu58/GBuF) using a 100-way alignment of 99 vertebrate genomes with the human genome to identify highly conserved CpG sites. To highlight this, we added to the introduction: “**Arrays used to build these clocks, including the HorvathMammalMethylChip40 developed by Arneson et al[14], were specifically designed for cross-mammalian application using conserved CpG sites selected through multi-species alignments** ” (Lines 52-55)
- We edited Supplemental Table 11 (annotations of significant CpGs from the SOS DMA) to include both cheetah (Aci_jub_2) and domestic cat (FC.9.0.100) annotations in addition to the original human (Hg38) annotations. These annotations were part of the cheetah annotation sheet that accompanied the manifest file from The Clock Foundation. Of note: these cheetah annotations were from the 2018 reference genome Aci_jub_2 RefSeq (GCF_003709585.1) which is currently suppressed for genome annotation processing. We believe providing both the feline and cheetah gene annotations alongside the human annotations will enhance the interpretability of our results and support future homology-based analyses.

Data Availability: A link to the raw data is missing. For transparency and reproducibility, which is a key policy for PLOS ONE, please provide a link to a public repository where the data are available.

- Our team is currently working to obtain the necessary permissions to upload the raw IDAT files, SESAME-normalized data received from The Clock Foundation, and the associated sample metadata sheet to NCBI GEO. We will provide the accession number once it becomes available.

Specific Comments

Abstract:

Please remove non-standard abbreviations from the abstract to adhere to journal guidelines.

- Reworded so that differential methylation analysis (DMA) abbreviations were removed (Lines 28 and 30). Kept abbreviations for sinusoidal obstruction syndrome (SOS) to succinctly describe the case-control analysis done (Lines 30-31).

L24: I suggest changing the terminology from “over the age of maturation” to a more standard term like “adult” individuals, and specifying the age threshold used (e.g., age > 2 years).

- Edited for clarity: “When applied to a test set of blood collected from live cheetahs, the clock provided accurate predictions for **adult individuals (age > 3 years)** but was less precise at and around age of sexual maturity” (Lines 24-25)

L30-33: The connection between these lines and the preceding sentences is unclear. Please revise to improve the flow of logic.

- Reworded to connect the disease analysis to the age and sex analysis: “**To explore the potential of methylation as a biomarker beyond age and sex**, we conducted a differential methylation analysis to investigate disease-related methylation patterns in cheetahs diagnosed with hepatic sinusoidal obstruction syndrome (SOS). This analysis identified 4,377 CpG sites with significant differences between SOS-positive and SOS-negative cheetahs (adjusted p-value < 0.05). These findings advance the development of epigenetic clocks for precise age and sex prediction in cheetahs and related species and establish a foundation for leveraging methylation biomarkers to investigate diseases in wildlife conservation efforts.” (Lines 27-33)

Introduction:

L44: Please specify whether the epigenetic modifications mentioned correlate positively or negatively with age in this context.

- Changed to indicate site-specific (can be up or down depending on site): “DNA methylation is one epigenetic modification that demonstrates **site-specific** linear changes that correlate with age” (Line 44)

L44-46: For clarity and conciseness, I suggest removing the phrase “and commonly occurs in regions of DNA where these cytosine residues are..”.

- Removed the phrase “and commonly occurs in regions of DNA where these cytosine residues are”: “DNA methylation involves the covalent binding of methyl groups to cytosine residues. These regions are termed “CpG sites”, with the “p” denoting the phosphate linker between the two residues.” (Lines 44-46)

L47: Please edit "methyl groups" to the standard form "methyl-groups".

- Changed “methyl groups” to “methyl-groups”: “The addition of **methyl-groups** results in structural changes to DNA that can affect gene expression” (Line 47)

L48: Please clarify what is meant by “structural changes” in this context.

- Reworded and added an example of how methylation can structurally block transcription: “The addition of methyl-groups results in structural changes to DNA that can affect gene expression. For example, methylation at transcription factor binding sites can block transcription initiation[[8]](https://paperpile.com/c/mdfZo3/r5kPe).” (Lines 46-48)

L49: A reference is missing.

- Added paper by Lu et al (reference #13) “Universal DNA methylation age across mammalian tissues” (Line 50)

L51: Please define what is meant by "Pan-species" upon first use.

- Added definitions for both pan-species and pan-tissue for clarity: “Pan-species **(i.e., applicable across multiple species) and pan-tissue (i.e., applicable across multiple tissue types)** clocks such as the universal pan-mammalian epigenetic clocks developed by Lu et al[[13]](https://paperpile.com/c/pmRb9u/0qQZs) provide versatile tools for cross-species comparisons” (Lines 50-52)

L61-62: I suggest rephrasing “tools” to something more precise, such as “these traditional age-estimation methods are difficult to apply accurately”.

- Reworded for clarity: “ Currently, skull measurements, radiographs, and dental examinations are the most common vertebrate **age estimation methods**[[17–20]](https://paperpile.com/c/pmRb9u/y2mNW+po6JQ+Ulu5H+nwjUc)-- **however, these methods are often difficult to apply accurately and consistently at scale in field settings**.” (Lines 63-65)

L63: Please add "Measurements of" or "The study of" before "epigenetic aging" for logical clarity.

- Reworded for clarity: “**Epigenetic age clock measurements** offer an alternative to these methods, with potential applications for determining not only chronological age but also health status.” (Lines 65-66)

L68: It is crucial information that all samples are from captive individuals with known chronological age, sex, and health status. Please state this clearly and early in the Introduction and Methods, as it is currently difficult for the reader to ascertain.

- Reworded to clarify age and sex was known for all SDZWA and MCDB samples (used to build the clocks): “Building epigenetic clocks for wildlife is challenging due to limited biobanked samples, especially blood. At the San Diego Zoo Wildlife Alliance, we leveraged liver samples from our biobank and cheetah blood profiles from the Mammalian Consortium Data Browser (MCDB), **all from captive individuals with known chronological age and sex**, to construct an epigenetic clock applicable to less-invasive samples like blood and skin (compared to liver).” (Line 69)
- Health status for MCDB samples not known but these samples were not used in health (SOS) analysis. Changed to specify health analysis done in SDZWA cheetahs: “Additionally, we used our extensive clinical and pathology database to investigate possible health status biomarkers **in San Diego Zoo-housed cheetahs**.” (Line 76)

The Introduction is missing an explicit mention of sex as a variable being investigated. Please incorporate this.

- Currently, the last paragraph mentions both age and sex as variables investigated: “ 1) investigate DNA methylation patterns to develop cheetah-specific epigenetic clocks for age **and sex** prediction and expand this across clinically and field relevant tissues and wild felid species” (Line 80)

L75-81: This section is the ideal place to formulate the clear hypotheses or research questions mentioned in the Major Revisions.

- Added clear hypotheses for the two specified goals: “We hypothesized that DNA methylation-based models could accurately predict age and sex in cheetahs, even with a limited sample size, and that incorporating multiple tissue types and related felid species would enhance predictive power and generalizability. Additionally, DNA methylation has been studied for its utility as a biomarker in various human and animal diseases [[25,26]](https://paperpile.com/c/pmRb9u/JJobB+yZNWA), and we hypothesized that epigenetic differences would distinguish SOS-affected and unaffected cheetahs” (Lines 84-89)

Methods:

L83: It would be very helpful to state explicitly at the beginning of this section that "All animals were born and raised in captivity and held under human care until death," or similar phrasing.

- Added for clarity: “The study population consisted of 447 cheetahs that had biobanked tissue samples available for use. **All animals were born and held under human care until death**.” (Line 96)

L89: Please change “die” to “died”.

- Changed to “died”: “Samples were collected as part of a complete necropsy performed routinely on all animals that **died** at our facilities.” (Line 99)

L91: Please clarify if n=7 refers to the number of samples or individuals.

- Changed to clarify that seven samples corresponding to seven individuals were used, and changed “blood” to “whole blood” to clarify sample type: “Biobanked **whole blood** samples **(n = 7)** collected from **seven** live cheetahs opportunistically during exams were also included” (Line 101).

L93-94: I suggest rephrasing; "depicts correlations" could be changed to "indicates repetitive sampling from the same individuals over time" if that is the intended meaning.

- Rephrased for clarity on repetitive sampling from same individuals: “Table 1 summarizes the tissue types used in the study, and **depicts repetitive sampling from the same individuals** (Table 1)” (Lines 104-105)

L103: Since two different extraction kits were used, please confirm whether a potential batch effect was investigated and controlled for.

- The clustering analysis performed on the samples didn’t show any distinct clustering based on the extraction kit used. We updated Supplemental Figure 1 to include trait bars that annotate sex, age, tissue, study and extraction kit to show this (image below), and edited the figure description in the manuscript to reflect these changes: “**D) Unsupervised hierarchical clustering of all cheetah samples used (outliers removed with the trait bars for annotation. Age spectrum is portrayed in red, with lighter red indicating younger age and darker red indicating older age. Sex is portrayed in pink and blue, with pink indicating female and blue indicating male. Tissue is depicted in red, orange and tan, with red indicating blood, orange indicating skin, and tan indicating liver. Study is portrayed in green and dark brown, with green indicating samples from SDZWA and dark brown indicating samples from MCDB. The DNA extraction kit used is indicated in blue and red, with blue indicating Qiagen extraction kit, red indicating NEB Monarch extraction kit, and grey indicating unknown (MCDB samples).**” (Lines 766-774)

- Additionally, we included a new methods section **“Clustering-based detection of sample outliers”** (Line 141) that explains the clustering analysis methods used (Lines 142-147).

L123: The description of the methods here is too brief; "converted using bisulfite sequencing and put through their previously published pipeline" is not well phrased and insufficient. Please provide a more thorough explanation of the steps involved.

- Created a new section in methods titled **“The Clock Foundation DNA methylation testing”** (Line 132) to distinguish what was done by The Clock Foundation (sample processing and testing on the array) and what was done by us (DNA extraction and data analysis on data returned to us from The Clock Foundation). The bisulfite conversion and sequencing lines were removed since we did not perform it, and the exact conversion kit and sample processing steps are not known (might be proprietary). The section now reads: “DNA was quantified on a Qubit 4 fluorometer (ThermoFisher) and approximately 250 ng of DNA was sent to The Clock Foundation (Torrance, CA) on dry ice overnight **for processing through their previously published pipeline for DNA methylation testing**[**[14]**](https://paperpile.com/c/pmRb9u/uKF6E)**. Briefly, the mammalian methylation array HorvathMammalMethylChip40 (Horvath Array) used to build methylation profiles from our genomic DNA samples was developed by Arneson et al**[**[14]**](https://paperpile.com/c/pmRb9u/uKF6E)**.**” (Lines 135-137)

L142: How was the sequencing performed, and how many samples were included in this step?

- The above changes should clarify that sequencing was performed by Clock Foundation, and we did the subsequent data analysis on the returned data. We added n counts to liver, skin and blood: “EWAS was performed separately on liver **(n =38)**, blood **(n = 7)** and **skin (n = 4)** samples using the R function standardScreeningNumericTrait from the WCGNA[[36]](https://paperpile.com/c/mdfZo3/5gOkT) R package to find DNA methylation changes that correlate with age using Pearson correlation test” (Lines 165-167)

L148: Please state the significance threshold (e.g., p-value) used for the analysis.

- Added the significance thresholds used for each analysis, making clear that a larger P-value cutoff was used for the smaller skin EWAS (explained further in results): “For liver and blood EWAS, p-value 10^-3^ was the significance threshold. For the smaller skin EWAS, p-value 10^-2^ was the significance threshold.” (Lines 167-169)

L153: Please consider adding a supplementary table listing the sample IDs, their known health status (especially for SOS), and other relevant metadata for the samples used in the final analyses.

- This is included in supplementary table S1, which lists relevant metadata including sample name, age, sex, SOS health status (and other liver classifications) and whether the sample was included in the SOS analysis.

L155-156: The sentence “SOS has been diagnosed in populations as early as 5 years old” is unclear. Does this refer to cheetah populations in general? Please rephrase for clarity. Also, please clarify how the age of these animals was determined.

- Rephrased for clarity: “**SOS was diagnosed in individuals from this cohort as early as 5 years old**, with suspect liver changes as early as 3 years old.” (Lines 178-179). Only SDZWA samples were used in the SOS DMA, so the age of the cheetahs at time of sample collection was documented.

L156-157: The current phrasing implies causality ("To capture potential epigenetic changes that lead to SOS..."). This is a strong claim. I suggest rephrasing to such as: “To identify potential epigenetic changes associated with SOS…”

- Rephrased as: “To identify potential epigenetic changes associated with SOS but avoid ASM bias, we limited samples to over 3 years of age (n = 30)” (Lines 179-180)

L158-165: This section describes various SOS states. For clarity, please focus only on the states that were actually included and analyzed in your study.

- Only SOS-positive samples were used for differential methylation analysis, but different states were annotated in the plots to visualize how different SOS states exhibited similar methylation (clustering together) at the CpG sites selected from the DMA. For clarity, added: “For the DMA, only samples with an explicit mention of SOS diagnosis (diagnosis of SOS) were classified as SOS-positive. **Additional categories ("SOS suspect", "non-SOS liver changes," and "no liver diagnosis") were annotated only in visualizations**.” (Lines 188-189)

L183: Please specify which software or online tool was used to perform the KEGG pathway analysis.

- Added R function and program, as well as citation to the sentence: “**Enrichment analysis was done using the function enrichKEGG from the R program clusterProfiler**” (Lines 209-210)

Results:

L189-196: This section appears to describe methods rather than results. Please consider moving it to the Methods section to maintain a clear distinction.

- Moved this section to Methods under **“Clustering-based detection of sample outliers”** (Line 141) that explains the clustering analysis methods used (Lines 142-147). Also moved the table with the final sample counts following outlier removal to this section in methods (Line 148)

L196-199: Please clarify why stillborn individuals are mentioned if they were not included in the analysis, or remove the mention if it is not relevant.

- We decided to remove the stillborn samples all together to avoid confusion (n=3, all cheetah liver samples). In the above section moved to Methods, the phrase “three were stillborn” was removed from Line 118: “These records documented a variety of health backgrounds: two cheetahs had no underlying disease, **three were stillborn (REMOVED)**, and the rest were reported to have one or more diseases at time of death, including bacterial infections, gastritis, chronic kidney disease, and sinusoidal obstructive syndrome (SOS)” (Line 118)
- Total sample counts were adjusted to reflect this in text: Lines 95, 97, 106, 109, and 117. Additionally, Table 1 (Line 108) with sample type counts was edited: total liver changes changed from 45 to 42.

L202: Please provide the justification for excluding outliers from the analysis.

- The new section in Methods **“Clustering-based detection of sample outliers”** (Line 141) now provides justification for exclusion based on clustering analysis (Lines 142-147).

L204: Please define "pan-tissue" in this context.

- Added definitions for both pan-species and pan-tissue to the introduction: “Pan-species **(i.e., applicable across multiple species) and pan-tissue (i.e., applicable across multiple tissue types)** clocks such as the universal pan-mammalian epigenetic clocks developed by Lu et al[[13]](https://paperpile.com/c/pmRb9u/0qQZs) provide versatile tools for cross-species comparisons” (Lines 50-52)

L216: Do the "blood" samples refer to whole blood or a specific fraction (e.g., PBMCs)? If whole blood was used, please discuss the potential confounding effect of variable blood cell composition, particularly in the context of disease or infection.

- Added to discussion a paragraph on the use of whole blood in this study, highlighting limitations and discussing how future studies could incorporate blood composition analyses or cell sorting to potentially improve age models, citing a study by Houseman et al (reference #47) on estimating cellular heterogeneity in whole blood samples from DNA methylation data: “Additionally, our test set included biobanked whole blood samples rather than isolated cell populations. Variation in blood cell composition, particularly in individuals with subclinical disease or immune activation, may have introduced methylation variability and affected prediction accuracy [[47]](https://paperpile.com/c/pmRb9u/DTvv). Future studies may benefit from incorporating blood cell composition analysis or using sorted cells to improve disease and age models.” (Lines 443-447)

L231: Please use the abbreviation LOOCV consistently after it is first defined.

- After this initial definition in Line 226, edited the following lines to use the abbreviation of LOOCV: Line 242, Line 253, and Line 276.

L246-248: In the text, please specify the sample sizes for each group in this analysis (e.g., N=x males, N=y females; N=x liver, N=y blood).

- For clarity, edited the sentence to include n counts for each sex for each tissue and species type tested, and rephrased (added “the same”) to show that the sex analysis was done on the same sample set as the age analysis: “A CheetahClock for sex was trained on **the same** combined SDZWA liver and MCDB blood data and used 67 CpG sites **(male n = 16, female n = 31)** (Supplemental Table 5-6). Sex was accurately predicted in the seven blood **(male n = 5, female n = 2)** and four skin **(male n = 2, female n = 2)** test samples , as well as in all tiger **(male n = 5, female n = 4)** and lion **(male n = 1, female n = 6)** samples from MCDB (Supplemental Table 7).” (Lines 257-261)

L249: Please revise the section title to be more descriptive of the results found.

- The section titles were revised to better reflect the results found. “UniversalClock and CatClock performance on cheetah samples” (Line 214) was used to head the results of our data tested on previously developed clocks, “CheetahClock performance across tissues and related felid species” (Line 224) was used to head the results of the CheetahClock we built and tested, and “FelidClock performance across felid species” (Line 263) was used to head the results of the FelidClock we built and tested.

Discussion:

L310: Please ensure the terms "hypomethylation" and "hypermethylation" are used consistently throughout the manuscript and that it is always clear which group is being referenced (e.g., "hypomethylated in SOS-positive individuals compared to controls").

- Edited for clarity: “This pilot differential methylation SOS analysis revealed 3,564 hypomethylated and 705 hypermethylated significant CpG sites in SOS-positive individuals compared to SOS-negative individuals.” (Lines 344-345)

L401: This is a good point. Please expand the discussion of accelerated aging in the context of the cheetah's life history traits.

- Expanded on discussion accelerated aging, citing two metabolic studies by Maly et al (references #42 and #43) on the physiological and metabolic changes female and male cheetahs undergo during pubertal transition: “The log-linear transformation used in the CheetahClock to account for increased methylation around ASM may not fully capture the non-linear dynamics of methylation changes occurring during this developmental period, which could lead to age prediction errors in individuals approaching or reaching ASM. **Metabolic studies of captive cheetahs have shown they exhibit a rapid, nonlinear phase of physiological development as they approach sexual maturity (roughly 1.5-3 years old)** [**[42,43]**](https://paperpile.com/c/pmRb9u/azevo+o2MuT)**. For example, male cheetahs reach adult weight by around 21 months, and their testosterone concentrations increase to reach adult levels by 18-24 months** [**[43]**](https://paperpile.com/c/pmRb9u/o2MuT)**. Female cheetahs likewise attain adult body weight around 21 months, and between 24-30 months their estrogen levels increase and they begin ovarian cycling** [**[42]**](https://paperpile.com/c/pmRb9u/azevo)**. In addition to these sex hormone shifts, glucocorticoid levels also become elevated during this pubertal transition** [**[42,43]**](https://paperpile.com/c/mdfZo3/jwOfT+Sf0Li)**. These nonlinear developmental trajectories suggest that DNA methylation may also change rapidly and irregularly during this life stage.**” (Lines 419-429)

L409: Suggest changing “under” to “before”.

- Changed “under” to “before”: “Other epigenetic clock studies have found deviations from log linear transformation, such as square root transformation in primates, leads to better predictions in animals **before** ASM[[44]](https://paperpile.com/c/pmRb9u/HRBnP)” (Line 431)

L411: The lines state, that “Methylation dynamics are often exponential.” Please elaborate on how the results presented in this study support this statement.

- Added a sentence linking our findings to the statements on exponential/non-linear methylation dynamics: “**Our findings support these patterns, particularly around ASM, suggesting methylation changes occur more rapidly or irregularly during early adulthood**.” (Lines 434-436)

L412-417: The observed differences around the age of sexual maturation (ASM) could potentially be confounded by the sex of the animals. Please consider and discuss this possibility.

- In general, these studies would benefit from having more samples from individuals around ASM to capture these trends. Added a sentence to highlight how sex could influence non-linear aging patterns around ASM, and how future studies can take this into account: “**It is also possible that sex-specific differences in pubertal timing contribute to the observed overestimations around ASM, particularly if methylation shifts occur earlier or more abruptly in one sex.** Expanding our approach to account for these shifts, possibly by incorporating a stage- **and sex-**based classifier and adding additional samples to our training set around ASM, could better capture the biological complexities of aging and improve prediction accuracy for younger individuals.” (Lines 437-440)

L425: Were skin samples taken from the same anatomical region on all animals? Different body parts may be exposed to different levels of environmental factors and could exhibit different aging rates. Please clarify or discuss this as a potential limitation.

- Our pathologists here at SDZWA said skin samples are typically collected from the axial/trunk/abdomen usually, but we can’t be 100% sure where these particular samples were taken from. To address this limitation and advise on future procedures, Lines 456-460 were edited:: “**Although skin biopsies were typically collected from the trunk or abdomen in these necropsy cases, we cannot confirm that all samples originated from the same anatomical region. Variation in sampling location could introduce additional variability, as skin from different body parts may experience different environmental exposures or aging rates.** Incorporating more **anatomically consistent** skin samples into future studies would capture more tissue-specific methylation patterns, potentially improving the MAE while maintaining high correlation.” (Lines 456-460)

L507: The sentence ending is incomplete ("hypomethylated in xx"). Please complete it.

- Edited the sentence to reference comparison to SOS-negative samples: “The DMA and heatmap analysis revealed that most CpGs implicated in SOS-positive samples were hypomethylated **compared to SOS-negative samples**, while some sites exhibited distinct hypermethylation.” (Line 456)

**Additional edits:**

- Added an acknowledgement to our Conservation Genetics team at SDZWA in the Acknowledgments section (Line 591)
