# Supplementary material for: Investigating epigenetic biomarkers of age, sex, and disease in captive South African cheetahs (Acinonyx jubatus jubatus)
Source: PLoS One. 2026 Jan 13;21(1):e0336127. doi: 10.1371/journal.pone.0336127 (PMC12798976; doi:10.1371/journal.pone.0336127)
Supplement: Attachment — Submitted filename: Response to Reviewers_2.docx [file pone.0336127.s020.docx]

Dear Reviewers and Editors,

We thank you for your thoughtful and constructive feedback with the second round of comments. We appreciate your continued interest in this paper.

The two main concerns are 1) data availability and 2) methods from The Clock Foundation service we used:

1) Raw IDAT files, SESAME-normalized data from The Clock Foundation, and the sample metadata sheet are ready for submission to GEO. However, the GEO submission sheet requires fields to be filled out for the submission, detailing their conversion process and protocols used (labeling, hybridization, scanning protocols). We have contacted them for this information, and they let us know they are working on it, but they are taking a while. In the meantime, we have uploaded the IDATs, the IDAT metadata file with the protocol information we were able to fill out (extraction and normalization methods), and the normalized data file as a zipped folder (IDATS_normalized_data) for submission so the normalized data used in the study is available for use.
2) We have updated the manuscript to include more information on the Clock Foundation methods using information available to us. Please see below for more details.

In the manuscript text we incorporated reviewer-recommended references and updated supplementary material titles and descriptions. The renamed supplementary files were re-uploaded to match PLOS ONE style requirements. All edits are described in detail below in red text with line numbers referencing the revised manuscript.

Thank you again for your time and advice.

Sincerely,

Michelle Ysrael, Steven Kubiski, and Caroline Moore

**---------------------------------------------------------------------------------------------------------------------------------------------**

Reviewer's Responses to Questions

**Comments to the Author**

1. If the authors have adequately addressed your comments raised in a previous round of review and you feel that this manuscript is now acceptable for publication, you may indicate that here to bypass the “Comments to the Author” section, enter your conflict of interest statement in the “Confidential to Editor” section, and submit your "Accept" recommendation.

Reviewer #1: All comments have been addressed

Reviewer #2: All comments have been addressed

2. Is the manuscript technically sound, and do the data support the conclusions?

The manuscript must describe a technically sound piece of scientific research with data that supports the conclusions. Experiments must have been conducted rigorously, with appropriate controls, replication, and sample sizes. The conclusions must be drawn appropriately based on the data presented.

Reviewer #1: Yes

Reviewer #2: Yes

3. Has the statistical analysis been performed appropriately and rigorously?

Reviewer #1: Yes

Reviewer #2: Yes

4. Have the authors made all data underlying the findings in their manuscript fully available?

The [PLOS Data policy](https://urldefense.com/v3/__http://track.editorialmanager.com/CL0/http:*2F*2Fwww.plosone.org*2Fstatic*2Fpolicies.action*23sharing/1/010f0199199a58b2-cadf207c-b3dc-41d3-afaa-6c7bb38fb8bb-000000/hi6ZfIZtgnYoDdpuBVAGGTQ6AJlDLd_LBVhO-gcCRTk=227__;JSUlJSU!!D3gL-16rZ1Oa!3cE0RtnSGzwJOxmEJnuwnUe4YG5cGzeFmJyyPij0Hl2JYhNg2LS1-esLugrfHydnyclfj01dYtrFGIK_5iy0ZvmL$) requires authors to make all data underlying the findings described in their manuscript fully available without restriction, with rare exception (please refer to the Data Availability Statement in the manuscript PDF file). The data should be provided as part of the manuscript or its supporting information, or deposited to a public repository. For example, in addition to summary statistics, the data points behind means, medians and variance measures should be available. If there are restrictions on publicly sharing data—e.g. participant privacy or use of data from a third party—those must be specified.

Reviewer #1: No

Reviewer #2: No

5. Is the manuscript presented in an intelligible fashion and written in standard English?

PLOS ONE does not copyedit accepted manuscripts, so the language in submitted articles must be clear, correct, and unambiguous. Any typographical or grammatical errors should be corrected at revision, so please note any specific errors here.

Reviewer #1: Yes

Reviewer #2: Yes

6. Review Comments to the Author

Please use the space provided to explain your answers to the questions above. You may also include additional comments for the author, including concerns about dual publication, research ethics, or publication ethics. (Please upload your review as an attachment if it exceeds 20,000 characters)

Reviewer #1: The authors addressed most of my comments. Please provide a GSE number in the data availability section.

Raw IDAT files, SESAME-normalized data from The Clock Foundation, and the sample metadata sheet are ready for submission to GEO. However, the GEO submission sheet requires fields to be filled out for the submission, detailing their conversion process and materials used. We have contacted them for this information, and they let us know they are working on it, but they are taking a while. In the meantime, we have uploaded these files as a zipped folder for submission so the normalized data used in the study is available for use.

Reviewer #2: Dear authors,
thank you for addressing my comments.
Most of them have been solved sufficiently.
The onces missing concern the reproducibility of your results:

1) the data upload is not yet completed, as you wrote: "Our team is currently working to obtain the necessary permissions to upload the raw IDATfiles, SESAME-normalized data received from The Clock Foundation, and the associated sample metadata sheet to NCBI GEO. We will provide the accession number once it becomes available."

Please finalise before acceptance.

Raw IDAT files, SESAME-normalized data from The Clock Foundation, and the sample metadata sheet are ready for submission to GEO. However, the GEO submission sheet requires fields to be filled out for the submission, detailing their conversion process and materials used. We have contacted them for this information, and they let us know they are working on it, but they are taking a while. In the meantime, we have uploaded these files as a zipped folder for submission so the normalized data used in the study is available for use.

2) The method used for estimating the epigenetic clock has not been more described as I asked for, but less. Other authors will thus not been able to understand their coming about, nor to reprodcue the data. Please try to explain what has been done for your samples, at least by summarising the methods from the paper you mentioned.

We hope the below helps clarify the steps taken to reach our results, while also highlighting the fact we used a service (The Clock Foundation) to generate the data and therefore cannot overstep the methods by making assumptions on what they did.

For building the clock models: Steps include 1) DNA extraction and shipping, 2) bisulfite conversion, 3) running a methylation array, and 4) data analysis. As mentioned, we performed steps 1 and 4, while the service company The Clock Foundation did steps 2 and 3. Since we do not know which kits they used and their exact methodology, we can only cite what the company cites for their steps.

- Step 1) Sample storage, DNA extraction and DNA quantification is covered in lines 114-117 and 134-138. We have added “DNA was quantified on a Qubit 4 fluorometer (ThermoFisher) and **plated on a fully skirted, low profile PCR style plate sealed with adhesive PCR plate foil seal (ThermoFisher). Samples were placed in random order to prevent any bias based on the chip**. Approximately 250 ng of DNA was sent to The Clock Foundation (Torrance, CA) on dry ice overnight for processing through their previously published pipeline for DNA methylation testing[[14]](https://paperpile.com/c/mdfZo3/fJGFF)*”* to the description of what was sent to The Clock Foundation.
- Step 2-3) The Clock Foundation then completes bisulfite conversion of the whole sample, followed by amplification and interrogated by the HorvathMammalMethylChip40 which covers up to 36k CpGs per mammalian species based on targeted CpG probes developed by Arneson et al [14]. These chips are read by an iScan (Illumina) and the raw data is sent back to the client with some quality control details. This section now reads “Approximately 250 ng of DNA was sent to The Clock Foundation (Torrance, CA) on dry ice overnight for processing through their previously published pipeline for DNA methylation testing[[14]](https://paperpile.com/c/mdfZo3/fJGFF). **Briefly, the Infinium method uses sodium bisulfite conversion and microarray-based single-base genotyping to assess CpG methylation: unmethylated cytosines are deaminated by bisulfite and read as thymines after PCR, and methylated cytosines are protected by conversion and remain cytosines. This platform enables high-throughput, multiplexed analysis using bead-bound probes and fluorescent single-base extension to distinguish cytosine to thymine conversions at target CpG sites[14]**. The mammalian methylation array HorvathMammalMethylChip40 (Horvath Array) used to build methylation profiles from our genomic DNA samples was developed by Arneson et al [[14]](https://paperpile.com/c/mdfZo3/fJGFF). The array targets 37,492 CpG sites, 35,988 of which are well conserved across many mammalian species[[28]](https://paperpile.com/c/pmRb9u/BkuBz).”. Other papers that have used The Clock Foundation data have written this section to industry standards based on peer-reviewed publications, for example:

<https://www.nature.com/articles/s41598-025-86705-5#Sec10>

**DNA methylation data**

All DNA methylation data were generated using the mammalian Infinium array “HorvathMammalMethylChip40”1. By design, the mammalian methylation array facilitates epigenetic studies across mammalian species (including humans) due to its very high coverage (over thousand-fold) of highly-conserved CpGs in mammals.

The Infinium method is based on sodium bisulfite conversion of DNA and microarray-based genotyping of CpG sites using Infinium bead technology with single-base resolution. The advantage of the microarray platform is that it is user-friendly, it can be multiplexed, and it exhibits good agreement with other platforms’ DNA methylation measures. Specifically, the Infinium beads bear a 23-base oligo address to locate them on the BeadChip, and a 50-base probe. Probe sequences are complementary to specific 50 base regions of bisulfite-converted genomic DNA. The 3′ end of the probe harbors the methylated CpG site to be monitored. After the probe is hybridized to bisulfite-treated test DNA, a single-base extension adds a fluorescently labeled ddNTP to the 3′ CpG site. This lets the C to T change caused by bisulfite conversion to be "genotyped." The fluorescent signal is then measured and processed.

Out of all CpGs on the mammalian array, 20,150 CpGs map to a large fraction of the cetacean species’ genomes1. However, when building all of the clocks for this study using only mappable CpGs, we find that overall performance of the clocks (via cross-validation analysis) became mildly or substantially worse, and age predictions in some species became very inaccurate within the cetacean multi species clocks. Therefore, we elected to use all CpGs on the mammalian array in the building of the clocks presented. Genome coordinate information can be downloaded from our GitHub page (https://github.com/shorvath/MammalianMethylationConsortium) and the supplementary information in1. The chip manifest file can be found at Gene Expression Omnibus (GEO) at NCBI as platform GPL28271. The SeSaMe normalization method was used to define beta values for each probe24.

- Step 4) Data analysis is covered in the subsequent methods sections (Lines 149-219)

**From round 1 revisions:**

L123: *The description of the methods here is too brief; "converted using bisulfite sequencing and put through their previously published pipeline" is not well phrased and insufficient. Please provide a more thorough explanation of the steps involved.*

Created a new section in methods titled “The Clock Foundation DNA methylation testing” (Line 132) to distinguish what was done by The Clock Foundation (sample processing and testing on the array) and what was done by us (DNA extraction and data analysis on data returned to us from The Clock Foundation). The bisulfite conversion and sequencing lines were removed since we did not perform it, and the exact conversion kit and sample processing steps are not known (might be proprietary). The section now reads: “Approximately 250 ng of DNA was sent to The Clock Foundation (Torrance, CA) on dry ice overnight for processing through their previously published pipeline for DNA methylation testing[[14]](https://paperpile.com/c/mdfZo3/fJGFF). **Briefly, the Infinium method uses sodium bisulfite conversion and microarray-based single-base genotyping to assess CpG methylation: unmethylated cytosines are deaminated by bisulfite and read as thymines after PCR, and methylated cytosines are protected by conversion and remain cytosines. This platform enables high-throughput, multiplexed analysis using bead-bound probes and fluorescent single-base extension to distinguish cytosine to thymine conversions at target CpG sites[14]**. The mammalian methylation array HorvathMammalMethylChip40 (Horvath Array) used to build methylation profiles from our genomic DNA samples was developed by Arneson et al [[14]](https://paperpile.com/c/mdfZo3/fJGFF). The array targets 37,492 CpG sites, 35,988 of which are well conserved across many mammalian species[[28]](https://paperpile.com/c/pmRb9u/BkuBz).” (Lines 138-145)

L142: *How was the sequencing performed, and how many samples were included in this step?*

The above changes should clarify that sequencing was performed by Clock Foundation, and we did the subsequent data analysis on the returned data. We added n counts to liver, skin and blood: “EWAS was performed separately on liver (n =38), blood (n = 7) and skin (n = 4) samples using the R function standardScreeningNumericTrait from the WCGNA[36] R package to find DNA methylation changes that correlate with age using Pearson correlation test” (Lines 172-174)

7. PLOS authors have the option to publish the peer review history of their article ([what does this mean?](https://urldefense.com/v3/__https://track.editorialmanager.com/CL0/https:*2F*2Fjournals.plos.org*2Fplosone*2Fs*2Feditorial-and-peer-review-process*23loc-peer-review-history/1/010f0199199a58b2-cadf207c-b3dc-41d3-afaa-6c7bb38fb8bb-000000/k-rGrZaHCmfSNJVWK1X07uSi6Q8q-JFqK8jpOJNWy-0=227__;JSUlJSUl!!D3gL-16rZ1Oa!3cE0RtnSGzwJOxmEJnuwnUe4YG5cGzeFmJyyPij0Hl2JYhNg2LS1-esLugrfHydnyclfj01dYtrFGIK_5o-pElmp$)). If published, this will include your full peer review and any attached files.

If you choose “no”, your identity will remain anonymous but your review may still be made public.

**Do you want your identity to be public for this peer review?** For information about this choice, including consent withdrawal, please see our [Privacy Policy](https://urldefense.com/v3/__https://track.editorialmanager.com/CL0/https:*2F*2Fwww.plos.org*2Fprivacy-policy/1/010f0199199a58b2-cadf207c-b3dc-41d3-afaa-6c7bb38fb8bb-000000/z5AvP5yTFnCg7FttbdSnPoz901C48_vODSeGquGYNQU=227__;JSUl!!D3gL-16rZ1Oa!3cE0RtnSGzwJOxmEJnuwnUe4YG5cGzeFmJyyPij0Hl2JYhNg2LS1-esLugrfHydnyclfj01dYtrFGIK_5vC5fXlP$).

Reviewer #1: No

Reviewer #2: No

[NOTE: If reviewer comments were submitted as an attachment file, they will be attached to this email and accessible via the submission site. Please log into your account, locate the manuscript record, and check for the action link "View Attachments". If this link does not appear, there are no attachment files.]

While revising your submission, please upload your figure files to the Preflight Analysis and Conversion Engine (PACE) digital diagnostic tool, [https://pacev2.apexcovantage.com/](https://urldefense.com/v3/__https://track.editorialmanager.com/CL0/https:*2F*2Fpacev2.apexcovantage.com*2F/1/010f0199199a58b2-cadf207c-b3dc-41d3-afaa-6c7bb38fb8bb-000000/imwgkl0YG7aVrGTyx2xK_h-txxiqxP0Onbf5Zi9R2sY=227__;JSUl!!D3gL-16rZ1Oa!3cE0RtnSGzwJOxmEJnuwnUe4YG5cGzeFmJyyPij0Hl2JYhNg2LS1-esLugrfHydnyclfj01dYtrFGIK_5luKfGgI$). PACE helps ensure that figures meet PLOS requirements. To use PACE, you must first register as a user. Registration is free. Then, login and navigate to the UPLOAD tab, where you will find detailed instructions on how to use the tool. If you encounter any issues or have any questions when using PACE, please email PLOS at figures@plos.org. Please note that Supporting Information files do not need this step.
